# QUANTITATIVE SYNTHETIC POLYMER MASS SPECTROMETRY WORKSHOP: Gaithersburg, MD November 7–8, 2002

**DOI:** 10.6028/jres.108.008

**Published:** 2003-02-01

**Authors:** William E. Wallace, Charles M. Guttman, Scott D. Hanton

**Affiliations:** Polymers Division, Materials Science and Engineering Laboratory, National Institute of Standards and Technology, Gaithersburg, MD 20899-8541; Air Products and Chemicals, Inc., 7201 Hamilton Blvd., Allentown, PA 18195-1501

## 1. Introduction

Mass spectrometry is a rapidly evolving measurement technique for synthetic polymers. It holds the promise of providing not only absolute molecular mass distributions but also quantitative end-group and repeat-unit composition, and structural information such as branching and intramolecular loop formation. It serves as an invaluable complement to size-exclusion chromatography and light scattering in molecular mass determination, and to nuclear magnetic resonance and vibrational spectroscopy for structure determination. The realization of the full promise of quantitative mass spectrometry presents challenges to researchers in all fields of polymer characterization and was the central topic of this workshop.

This Workshop comes at a propitious time as Koichi Tanaka and John Fenn were awarded the 2002 Nobel Prize in Chemistry for their separate work on gas-phase macromolecular ion generation. Their citation reads, in part, “Mass spectrometry is a very important analytical method used in practically all chemistry laboratories the world over. Previously only fairly small molecules could be identified, but John B. Fenn and Koichi Tanaka have developed methods that make it possible to analyze … macromolecules as well.”

## 2. Format

The workshop brought together researchers, practitioners and laboratory managers from industry, academia, and government to present new results, discuss recent trends, and identify important problems in the area of quantitative mass spectrometry of synthetic polymers. The format for the 1½-day workshop consisted of 1 day of formal presentations, a full-symposium dinner to encourage personal interactions, and a half day of open discussion emphasizing problem-solving. During the open discussion participants presented specific measurement challenges they have encountered in their work and solicited suggestions from the workshop attendees. In a role reversal from the traditional seminar format the speakers asked questions and the audience provided answers. The first-day formal talks focused on challenges to quantitation, including:

- sample preparation and characterization
- chromatographic separations coupled to mass spectrometry
- chemistry of gas-phase ion creation
- ion mass separation and detection
- data analysis algorithms
- examples of specific synthetic polymers.

The open discussion session format consisted of each presenter giving a ten-minute description of a particular measurement challenge followed by a moderated discussion where participants offered measurement ideas and experiences relevant to the problem at hand.

## 3. Review of Workshop Presentations

### 3.1 Sample Preparation

The discussion of sample preparation focused on matrix-assisted laser desorption/ionization (MALDI) and whether or not sample homogeneity is a necessary condition for quantitation. The argument was made that target homogeneity, where the analyte is in intimate contact with the matrix (i.e., the analyte is “well solvated” by the matrix), is a necessary, but not sufficient, requirement for quantitative MALDI mass spectrometry of synthetic polymers. To this end the matrix+analyte+salt solution may be electrosprayed onto the sample holder while taking extra care to confirm that each of the three components are fully dissolved in the solvent at the concentrations employed. Electrospraying the solution at a moderately high voltage (typically 5 kV) from a thin steel capillary tube produces fine droplets of solution. These droplets fly in air, typically over a distance of about 3 cm to the target. In transit the solvent evaporates. This in turn means that getting this distance correct is important; however, it is different for each solution and for the specific spraying conditions used. What is deposited is a fine powder demonstrating intimate mixing between the three solid components. This gives good mass spectra with the advantage that the spectrum is the same at all points on the target, that is, “sweet spots” (defined as regions giving especially strong analyte signal) are eliminated. This mixing is facilitated by using non-polar matrixes with non-polar solvents, and polar with polar. Lastly, optimizing the matrix to analyte ratio as a function of analyte molecular mass was shown to be critical in obtaining quantitative results from the mass spectrum. This last point implies that broad polydispersity polymers may never be amenable to molecular mass distribution quantitation by MALDI mass spectrometry.

A contrary view was presented that reproducible, high-quality spectra could be generated by simply grinding together (in a mortar and pestle or with a ball mill) the matrix, analyte and salt to form a powder with particles on the scale of tens of micrometers in diameter. The degree in homogeneity obtained appears to be higher than with the solvent-based approach and excludes a prerequisite condition of an analyte embedded in the matrix crystal lattice. However, the relevant homogeneity length scale remains unknown. This was shown through specific examples to require lower laser power for desorption/ionization, which leads to milder MALDI conditions (e.g., less macromolecular fragmentation) as well as yielding highly reproducible spectra. These spectra had improved signal-to-noise ratio with a lower baseline than solvent-based methods using hand spotting. Both of these are requirements for successful quantitation. An added advantage of this method is that it requires no solvent and, thus, avoids difficulties with dissolving the analyte+matrix+salt mixture into a common solvent and, subsequently, removal of solvent prior to measurement (e.g., segregation). The added advantge is useful in cases where the analyte is insoluble. It was noted that this process is reminiscent of the early work of Koichi Tanaka mentioned in the Introduction. A key difference between Tanaka’s work and the results presented at the workshop is that Tanaka used fine cobalt metal powder, not the organic acids used predominantly today.

### 3.2 Chromatography and Mass Spectrometry

Often the separation of a wide-polydispersity polymer into more narrow mass fractions facilitates quantitation by mass spectrometry. Two talks, one on polyethylene oxide/polypropylene oxide/polyethylene oxide (PEO/PPO/PEO) triblock copolymers and another on polyamides, demonstrated the potential of this approach to quantitation. The PEO/PPO/PEO work relied on size-exclusion chromatography (SEC) to narrow the polydispersity of the polymer followed by time-of-flight (TOF) MS/MS for structural and molecular mass analysis. Between the two TOF separations the samples were passed through a helium-filled collision-induced dissociation cell. This three-stage analysis (SEC-MS-MS) was able to discern block length in the PEO/PPO/PEO triblock copolymer as well as the block length versus overall molecular mass. It was noted that higher mass oligomers are more difficult to fragment making their analysis more difficult. In the second talk polyamides with different end groups were separated by size-exclusion chromatography and by liquid adsorption critical chromatography (LACC). This was accomplished via a two-dimensional methodology so the molecular size could be separated from end group composition (SEC-MS and LCC-MS). After separation, MALDI-TOF mass spectrometry was used to identify the exact chemical composition of the end groups, and to correlate molecular size with molecular mass.

### 3.3 Ion Creation by Laser Desorption

Matrix-assisted laser desorption/ionization can best be described as a complex interplay of multiple processes. The first presentation compared a new quantitative model of ionization in ultraviolet MALDI with experiment. The interplay between the photochemical processes of ionization and the physical processes of desorption was found to be crucial to the formation of intact macromolecular ions in the MALDI process. Indeed much of this interplay was shown to occur in the expanding plume well after the laser pulse had ended. Primary ions (often matrix), which are formed early, react in the plume to form the thermodynamically most favorable products, which we observe at the detector. With respect to such secondary ion-molecule reactions, the plume is found to reach or approach equilibrium (depending mainly on plume density, a function of laser fluence). The thermodynamics of secondary reactions explains many known characteristics of polymer MALDI, and provide opportunities for further development. Since many polymers do not have high proton affinities (PAs), they compete poorly with matrix molecules for this ionization channel. On the other hand, although metal ion affinities (MAs) are much lower than PAs, polymers can in many cases effectively compete, and are thereby better ionized in this manner. This is reflected in the fact that empirically discovered “synthetic polymer” matrices (e.g., dithranol) have been found to have particularly low MAs. A fundamental difficulty in polymer MALDI is shown by the model to be caused by the magnitude of typical MAs (or more precisely the small difference between matrix and analyte MAs). This leads to a relatively low degree of reaction, so that polymer analytes are less completely and efficiently ionized than, for example, high PA biomolecules. The problem becomes worse as MAs decrease, as for example with saturated polymers lacking functional groups.

The next presentation took a different approach by using molecular dynamics calculations with the breathing-sphere model to visualize the ion creation process. The MALDI event can be divided into two regimes. The first, termed desorption, occurs at low laser fluence and is characterized by the ejection of single molecules, both matrix and analyte. The second, termed ablation, occurs at higher laser fluences and is characterized by the ejection of molecular clusters in a very dense plume with many intermolecular collisions. Ablation can be subdivided into two regimes itself. For longer laser pulses the ablation mechanism is controlled by thermal confinement and results in the ejection of interconnected liquid-like clusters that cool as material evaporates from them. For shorter laser pulses the ablation mechanism is controlled by stress confinement of the shock wave. This mechanism produces larger, faster moving clusters than in the thermal confinement regime. However, the total yield of individual molecules appears not to be sensitive to the irradiation conditions. Rather, total fluence seems to be the most important parameter for the successful MALDI experiment.

### 3.4 Ion Detection and Data Analysis

The detection efficiency for high mass ions by the microchannel plate detectors commonly employed remains a persistent problem. In time of flight (TOF) mass separation the higher the ion mass the slower it is moving when it reaches the detector (for a fixed acceleration voltage). At a sufficiently high mass these slow-moving ions will trigger the detector with a low probability or, in extreme cases, fail to trigger the detector entirely. This severe undercounting of high mass oligomers will take its toll on the ability to quantitate via mass spectrometry. Furthermore, such detectors are strictly ion counting, that is, they register nothing of the relative mass of the ion. It was noted that the invention of a mass sensitive detector would be a welcomed development for quantitative macromolecular mass spectrometry. It was suggested that neither the MALDI process nor the method of mass separation (e.g., TOF) were limiting factors to quantitative synthetic polymer mass spectrometry. The next presentation described current microchannel plate detector enhancements that are aimed at increasing their sensitivity to high mass ions without compromising the detector response time. These include small channels (diameters down to 2 µm) to decrease detector response time (critical in time of flight applications), and the improvement of detector coatings making them more sensitive, but not necessarily uniformly sensitive, to slow moving ions.

The final step in obtaining quantitative results from mass spectra is data analysis that is robust and self-consistent. The variety of software available in commercial instrumentation fails to meet these criteria. In consideration of these needs a nonlinear programming algorithm using an L_2_ approximation to L_1_ fits for rapid and repeatable processing of mass spectral data that may contain hundreds of peaks without operator intervention was described next. The flow of the algorithm is: 1) given a data set of *N* points we find a collection of *strategic points* and the unique optimal piece-wise linear function passing through the **x** coordinate of each strategic point, 2) this defines a new function from which the peak maxima and the peak limits can be easily determined, 3) the original data are then integrated under each peak maximum between the two adjacent minima. This method requires no knowledge of peak shape and no preprocessing of the data, i.e., smoothing or baseline correction. Lastly, it was demonstrated that the power spectrum of the noise in a typical MALDI-TOF mass spectrum cannot be predicted solely from the experimental conditions; and therefore, blind application of smoothing and/or filtering algorithms may unintentionally remove information from the data which is detrimental to quantitation.

### 3.5 Applications and Case Studies

The first application of quantitative synthetic polymer mass spectrometry presented involved the creation of the ideal molecular mass distribution as a test specimen for quantitative molecular mass distribution measurement. Supercritical fluid chromatography (SFC) was used to separate individual low mass polystyrene oligomers into distinct fractions. These oligomers where then mixed in equal molar ratios in groups of about five or six oligomers. In principle this should give a flat distribution. If it does not, then mass discrimination is present in the experiment. Tests can then be performed to find the source of this discrimination. For polystyrene oligomers, it was found that there was discrimination against the lower mass oligomers and that this discrimination worsened with increasing laser power. An ideal mass distribution could not be measured. Work continues to discover why.

The next two presentations covered quantitation of copolymer distributions. The first reported on the use of size-exclusion chromatography linked to either nuclear magnetic resonance or mass spectrometry. The combination of these three methods was able to provide unprecedented information on the copolymer distribution while maintaining intact oligomers. The second presentation took a different route. By purposefully fragmenting the copolymer molecules using collision induced fragmentation the lengths of the respective blocks can be deduced. It was noted that an understanding of gas phase unimolecular reaction (e.g., rearrangements) was critical in interpreting the mass spectra.

The final presentation of the first day dealt with the identification of polymer end groups from MALDI or electrospray ionization mass spectrometry and then using this information to deduce mechanistic details in order to improve synthetic routes and yields. More difficult questions were approached by MS/MS methods analogous to those used in the copolymer studies discussed earlier. It was noted that mass spectrometry could be sensitive to species at the 1 % mol fraction, well below what can be measured by nuclear magnetic resonance for the examples described.

## 4. Problem Solving Session

At the special session held on the second day workshop participants were asked to present measurement problems that they have encountered in their own work for comment and discussion by the other workshop participants. This resulted in a very lively working session. The problem-solving session served to build the synthetic polymer mass spectrometry community by providing a place to have difficult measurement questions addressed without compromising intellectual property issues.

The first topic was characterization of polyolefins, particularly polyethylene and polypropylene. In general these materials are difficult to analyze by chemical means due to their lack of heteroatoms and unsaturated bonds. Specifically, for mass spectrometry they are exceedingly difficult to form as intact, gas phase ions. Beyond this, the two leading measurement challenges for these materials by any analytical means are the determination of long chain branching characteristics (branch length as well as spacing between branch points) and the determination of copolymer sequences (both short and long run sequences). It was suggested that polymers with branches occurring in as low as 1 in 10 000 repeat units are important to the physical properties of the polymer. Light scattering, or SEC with both light scattering and viscosity detectors, can approach the branching question but it is time consuming and only yields indirect information via molecular size in solution. Comments from workshop participants centered on the use of pyrolysis gas-chromatography mass spectrometry to attack the copolymer problem. As for the branching challenge it was generally agreed that attacking the molecule at the branch points was the key to success but how to do this was far from clear. Wet chemical processes may work but at this time no one has been able to isolate a chemical method that selectively attacks the branch points. A suggestion was made that ultra-short laser pulses (resulting in very high field strengths) may break the molecule at the branch points.

The second topic centered on accurate mass measurements for the unique identification of end groups. Specifically, the questions that arose were: What accuracy and precision can be achieved by MALDI-TOF mass spectrometry on small oligomers and, Are these adequate to uniquely identify the chemical composition of the end groups? The observation was made that assuming the (unknown) mass uncertainty is constant across the mass spectrum, then the smaller the end-group mass is (relative to the mass of the oligomer) the larger is the uncertainty in the measured mass of the end group. Furthermore, since the degree of polymerization (i.e., number of repeat units) is frequently unknown for a given peak there opens up a large number of possible end group atomic compositions. How can these choices be narrowed? First, the analyst can use what is known of the chemistry and propose logical structures and eliminate highly unlikely structures. Second, at low mass the isotope distribution can be used to eliminate possible structures. It was interesting that even with rather large mass uncertainties, end group information can still be obtained by MALDI-TOF mass spectrometry.

The next presentations dealt with quantifying the relative amount of material in a mixture. For many materials there is a need for an internal standard that has a mass close to the material of interest but does not either charge preferentially or suppress the charging of the material of interest. The search for such an internal standard can be time consuming and the result can be less than satisfactory. If the material can be separated, chromatographically for example, then a calibration curve can be created. If this is not possible it would be best to run the mass spectrometer with no delayed extraction and in linear mode to decrease the chances the instrument is the source of discrimination. Lastly, it was suggested that a deuterated analog to one or more of the components of interest could be used as an internal standard. A similar presentation followed where it was observed that under certain electrospray ionization conditions specific oligomers in a mixture could be either enhanced or suppressed. It was suggested that for electrospray ionization the choice of solvents (single or multiple) is crucial to the successful experiment. These solvents must not only dissolve the analyte but must also dissolve and dissociate any added salts to insure an adequate supply of metal cations to ionize the polymer analyte. Furthermore, there may be a significant difference in the rate of ion production of cyclic versus linear homologs. A suggestion was made to use ammonia as the nebulizing gas for electrospray ion production in order to avoid issues with salt solubility. It was also suggested that a spray of two solutions (one with analyte and one with salt) from concentric capillary tubes has been found to be useful by some practitioners. Lastly, it was suggested that drying the solvents might be critical to proper electrospray technique.

The final presentation considered the effects of mating size-exclusion chromatography with mass spectrometry. The SEC measures molecular hydrodynamic size while mass spectrometry measures mass. Therefore, cyclics and hyperbranched molecules behave differently from linear molecules. For this reason you need to couple chromatography with mass spectrometry. It was noted that using an ultraviolet/visible detector (in addition to perhaps tagging the molecule with a chromaphore) gives superior quantitative results to the SEC. This type of detection is sensitive to the total mass of polymer in the oligomer while mass spectrometry is sensitive to the number of ions. Together they can answer general questions on molecular structure.

## 5. Summary and Conclusions

This workshop revealed that both the matrix-assisted laser desorption/ionization and the electrospray ionization methods are still poorly understood. Much work remains, both experimental and theoretical, to develop an understanding whereby the conditions that will, and those that will not, give quantitative results are thoroughly delineated. As one speaker commented, “the MALDI process cannot be understood through MALDI mass spectrometry experiments alone”. It is imperative that methods be brought in to understand how target composition and structure affect plume physics and chemistry, and how these factors ultimately determine how macromolecular gas phase ions are produced. More particularly, it must be elucidated under what conditions the MALDI (or ESI) process discriminates against certain analyte characteristics (e.g., molecular mass, chemical composition, molecular structure, etc.) for it is in this discrimination that quantitation will be gained or lost.

Detecting slow-moving macromolecular ions remains a challenge. The microchannel plate detectors currently in use have a severe roll off in sensitivity with ion velocity (i.e., mass in time-of-flight systems). Detectors based on other physics (e.g., cryogenic superconducting detectors) lack the rapid response and/or the sensitivity of microchannel plates. Finally, a detector that registered the momentum of an ion (i.e., a mass fraction detector) would be of great benefit to measure high mass ions in a distribution which may be few in number but significant in total mass. Chromatographic separations performed before the mass spectrometry may offer a route around these problems.

Questions of data reduction and spectrum analysis remain unresolved. Peak integration in the presence of noise and a non-zero baseline often requires operator judgment. This can bias attempts at drawing quantitative conclusions from data. At this time no generally agreed upon, robust method exists to overcome this challenge.

Many participants recommended that a follow-up conference be held in the fall of 2003 and that the time allotted for the open discussion be lengthened.
